# The European charter for counteracting obesity: A late but important step towards action. Observations on the WHO-Europe ministerial conference, Istanbul, November 15–17, 2006

**DOI:** 10.1186/1479-5868-4-11

**Published:** 2007-04-05

**Authors:** Johannes Brug

**Affiliations:** 1 EMGO Institute, VU University Medical Center, Van der Boechorststraat 7, 1081 BT, Amsterdam, the Netherlands

## Abstract

**Background:**

On November 15–17, 2006 the World Health Organization Regional Office for Europe organised a ministerial conference on counteracting obesity in the European region. Delegations from 48 countries met in Istanbul, Turkey. Observed by relevant nongovernmental organisations and expert temporary advisors, the European ministers adopted a charter on counteracting obesity. This charter states that countries within the European region should be able to show results in slowing down and stopping the obesity epidemic within the next 4–5 years, especially among children, and that the obesity prevalence trends should be reversed before 2015. To achieve this, the charter explicitly calls for action beyond health education: changes in the physical, political, informational and social environments are needed to facilitate a healthy energy balanced lifestyle.

**Discussion:**

The fact that all member states of WHO-Europe have now explicitly agreed on an ecological approach to fighting the obesity epidemic with a timeline for visible results is important. However, the charter does not explicate specific enough and measurable objectives for improvement, nor the means needed to reach these.

**Summary:**

The fact that all WHO-Europe member states have agreed on a charter that recognizes that counteracting obesity requires a multidisciplinary and ecological approach, with a timeline for improvements, is a late but important step forward for public health policy and practice across Europe. However, more specific tangible goals should now be set, the required means should be allocated, coordinated and immediate action should be implemented, and research to identify effective strategies should be encouraged and facilitated.

## Background

After several years of careful preparation, a ministerial conference on counteracting obesity was organized from November 15–17, 2006, by the World Health Organization regional Office for Europe (WHO-Europe). This conference brought together ministerial delegations from 48 countries in the European region. The conference was held in the European part of the city of Istanbul, Turkey, with Asia only a crossing of the Bosporus away. This was an appropriate place for a conference specifically aimed at the European region, but with outcomes that are also relevant for other regions of the world.

The conference aimed to put obesity on the political and policy agendas, to come to some sort of consensus about the appropriate way and realistic timeline to reverse the obesity trends, and to encourage international and intersectoral collaboration for concrete action. Apart from the ministerial delegations, the approximately 600 attendees included representatives of various (international) nongovernmental organisations (NGOs) and mass media, as well as a number of key experts who were appointed as temporary advisors.

The preparation of the conference was a lengthy and careful process. This process was necessary to design, develop and reach consensus about a draft European Charter on Counteracting Obesity that was finalised and signed during the conference. This preparation process included several preparatory meetings with governmental representatives and nongovernmental experts; one preparatory expert meeting was held as a satellite to the fourth annual meeting of the International Society of Behavioral Nutrition and Physical Activity, in Amsterdam, the Netherlands, June 18, 2005.

The official highlight of the conference was the signing of a charter on counteracting obesity [1] by the director of WHO Europe and the chair of the conference, the Turkish minister of health, on behalf of all 53 member states of WHO-Europe. This charter first of all explicitly recognizes the seriousness of the obesity epidemic and the related health problems. The charter literally states that obesity "is one of the most serious public health challenges in the WHO European region", and it puts a special emphasis on the rising trends of overweight and obesity among children across Europe. The charter also recognises that the obesity epidemic has a negative impact on social and economic development. This implicates that fighting obesity is a challenge that should include other sectors than the public health field.

The charter does more than establishing international consensus on the problem analysis. It also includes broad priorities for action. Notably, the charter states that the obesity epidemic is a modifiable determinant of health: "It is possible to reverse the trend and to bring the epidemic under control". However, this will require changes in people's lifestyles that should be promoted by changes in personal motivations and choices, but also by changes in the social, physical, economical and political environments so that healthy, energy-balanced choices become better available, accessible and normative. The charter thus makes explicit that fighting the obesity epidemic requires societal and political actions, and that individuals alone cannot be held responsible for their obesity. In line with appeals by experts [2-4], the charter therefore asks for coordinated collaborations between governments, nongovernmental organisations, private enterprise, professional networks, and the media. These collaborations should go beyond the public health sector and should include sectors such as agriculture, sports, infrastructure, transport, marketing, tourism and entertainment. Actions are called for on an international, national and local level, and these should focus on both sides of the energy balance: physical activity and dietary intake.

The charter further provides a timeline for significant change. Within the next five years progress should be made in changes in the obesity trends in most countries of the European region, especially for children and adolescents; within the next ten years, before 2015, the obesity epidemic should have been reversed.

## Discussion

At the WHO Europe ministerial conference on counteracting obesity hardly any new facts were presented. The problems identified will be common knowledge and the avenues for action made explicit in the charter will probably not go far enough for experts in the field of obesity research or behavioural nutrition and physical activity. This conference was not aimed at providing or communicating new evidence about the obesity epidemic or effective intervention strategies. It was meant to formalize the consensus across the entire European region on development and implementation of evidence- and expert-based best-practice policy approaches to contribute to tackling the obesity epidemic in that region. The conference and the charter come rather late: the obesity epidemic has been underway for a few decades, and more timely action was possible. Nevertheless, I do believe that this international conference has been a significant step forward in tackling the obesity epidemic. However, it is only one first step on a road with many possible obstacles. The charter has been signed by all member states of WHO Europe. However, signing the charter will not lead to lower obesity prevalence. Actions based on the charter are now needed, and the states that signed the charter now have an obligation to initiate, encourage and implement real action to effectively curb the obesity trends.

Why is this charter an important step in the right direction?

### Environment next to individual responsibility

The charter states that individuals alone cannot be held responsible for their obesity, and that the societal problem of overweight and obesity is a joint responsibility of governments, civil society, and the private sector. Although experts in the field have adopted this ecological determinism model of the present-day obesity epidemic quite some time ago [5-7], European governments have not always accepted this approach. Even at the ministerial conference representatives of different countries especially stressed the personal responsibility of citizens to make healthful choices in their public statements. Putting such an emphasis on personal choice cleared governments of their responsibility to take the lead in obesity prevention. The fact that the charter, signed on behalf of all countries in the European region, highlights the interactions [8,9] between the individual choices of citizens and the obesogenic environment in promoting unnecessary weight gain, indicates that governments now recognise their responsibility to foster environments that provide better opportunities for energy-balanced lifestyles [10].

### Inclusion of other policy areas next to public health

The charter also recognises the fact that, in line with the aforementioned social-ecological model of obesity, different departments of national and local governments should join in obesity prevention efforts. If governments take their responsibility for promotion of obesity preventive environments seriously, national and local government departments responsible for policies on the physical environment (agriculture, urban planning, education), social-cultural environment (education, social affairs, culture, sport), economic environment (finance), and informational environment (education, consumer affairs, media) should join the Health Departments in creating an environment that motivates and enables energy-balanced nutrition and physical activity habits.

### Recognition of key determinants of obesity risk

The charter also identifies a few more specific important determinants of weight gain and obesity that should be targeted. Explicitly pointing a finger to certain causes of the obesity epidemic certainly helps to identify priorities for action. Marketing to children of energy-dense foods, high fat and free sugar contents of foods are mentioned as specific areas that need swift action.

### Focus on both sides of the energy balance

Not surprisingly, the charter states that changes are needed at both sides of the energy balance, i.e. in nutrition and in physical activity behaviours.

Why does the charter not go far enough?

### No SMART objectives

The goals stated in the charter are vague. It states that "visible change" should be achieved, in "most member states", and that the obesity trend should be "reversed". In order to evaluate if this charter indeed results in relevant changes, the charter's action plans should be translated into Specific, Measurable, Attainable, Realistic and Tangible (SMART) goals, and an evaluation framework to monitor progress towards these goals should have been put in place.

### Governments should take the lead and swift action is needed

An impressive consortium of NGOs (European Public Health Alliance, Consumers International and the European Consumers Organisation, the European and International Associations for the Study of Obesity, the European Childhood Obesity Group, the International Obesity task Force, the European Heart Network, the Federation of European Cancer Societies, the Federation of European Nutrition Societies, the International Sport and Culture Association, the International Baby Food Action Network, and the Royal College of Physicians of London), put out a press release during the conference. In this press release these NGOs congratulated WHO-Europe and the participating governmental delegations on the charter. However, they also asked for more immediate and concrete actions from governments. The consortium of NGOs explicitly stressed that effectively reducing obesity requires actions across all government departments with international coordination. Evidence-based concrete actions that the NGOs suggest include restricting marketing to children of foods high in fat, sugar and salt, better and easy to understand labelling of foods, establishing and enforcing food standards in schools, exploring fiscal or other financial measures to improve accessibility to healthy and reduce accessibility to less healthy foods, to increase the number, duration and intensity of physical education school hours and extra-curricular physical education, and to increase the opportunities for walking and cycling in urban settings (see also [9]. The NGOs also rightfully state that actions by the private sector are necessary for effective obesity prevention, but that a full reliance on public-private partnerships and self-regulatory, voluntary changes in the private sector is probably not enough to realise swift necessary changes in, for example, energy density, fat and sugar contents of foods, portion sizes, and the way food is marketed to children. A strong internationally supported governmental guidance and government enforced regulation is needed to speed up the process, to avoid lengthy negotiations and ineffective compromise actions. Such an approach requires strong political leadership.

A first follow-up initiative on the Istanbul conference and the charter is already underway. WHO Europe has started to prepare a second action plan for food and nutrition policy to promote and facilitate implementation of the good intentions addressed in the charter on counteracting obesity.

## Summary

• The charter on Counteracting Obesity signed on behalf of all 53 member states of the WHO-Europe region is an important step toward effective action to curb the obesity epidemic

• However, the charter does not go far enough in identifying explicit goals and means to effectively reverse obesity trends

• The charter should now be translated and specified in actions involving a range of policy areas, involving international coordination, national, regional and local governments, the private sector as well as the civil society.

## References

[B1] WHO European Charter on counteracting obesity.

[B2] Seidell JC, Nooyens AJ, Visscher TL (2005). Cost-effective measures to prevent obesity: epidemiological basis and appropriate target groups. Proc Nutr Soc.

[B3] Robinson TN, Sirard JR (2005). Preventing childhood obesity: a solution-oriented research paradigm. Am J Prev Med.

[B4] Booth KM, Pinkston MM, Poston WS (2005). Obesity and the built environment. J Am Diet Assoc.

[B5] Egger G, Swinburn B (1997). An "ecological" approach to the obesity pandemic. BMJ.

[B6] Hill JO, Wyatt HR, Reed GW, Peters JC (2003). Obesity and the environment: where do we go from here?. Science.

[B7] Ball K, Timperio AF, Crawford DA (2006). Understanding environmental influences on nutrition and physical activity behaviors: where should we look and what should we count?. Int J Behav Nutr Phys Act.

[B8] Kremers SPJ, de Bruijn GJ, Visscher TLS, van Mechelen W, de Vries NK, Brug J (2006). Environmental influences on energy balance-related behaviors: A dual-process view. Int J Behav Nutr Phys Act.

[B9] Brug J, van Lenthe FJ, Kremers SPJ (2006). Revisiting Lewin: How to Gain Insight in Environmental Correlates of Obesogenic Behaviors. Am J Prev Med.

[B10] Brug J, Oenema A, Ferreira I (2005). Theory, evidence and Intervention Mapping to improve behavioral nutrition and physical activity interventions. Int J Behav Nutr Phys Act.

